# Combined Endoscopic and Laparoscopic Treatment of Gallstones and Bile Duct Stones

**DOI:** 10.1155/DTE.5.223

**Published:** 1999

**Authors:** A. Longhini, R. Pellegrino, A. R. Kazemian, P. Borelli, G. Munarini, E. Buccilli, G. Marcolli

**Affiliations:** Department of General Surgery Civil Hospital via Stelvio 25 Sondrio 23100 Italy

## Abstract

The best treatment of bile duct lithiasis in the era of the laparoscopic cholecystectomy has not yet been defined, as we can choose between different kinds of therapies which range from the traditional laparotomic treatment to the more modern endoscopic and laparoscopic techniques. Although in some centres a wholly laparoscopic access to the bile duct is used, we believe that the fastest, safest and most reliable method is still the sequential treatment, endoscopic and laparoscopic. In our experience the endoscopic cleaning of the choledochus was performed in 87.1% of the cases, with no mortality and 4.2% morbidity.


*Diagnostic and Therapeutic Endoscopy*, Vol. 5, pp. 223–229
Reprints available directly from the publisher
Photocopying permitted by license only

© 1999 OPA (Overseas Publishers Association) N.V.
Published by license under
the Harwood Academic Publishers imprint,
part of The Gordon and Breach Publishing Group.
Printed in Malaysia.

### Combined Endoscopic and Laparoscopic Treatment of Gallstones and Bile Duct Stones

A. LONGHINI*, R. PELLEGRINO, A.R. KAZEMIAN, P. BORELLI, G. MUNARINI, E. BUCCILLI and G. MARCOLLI

*Department of General Surgery, Civil Hospital, via Stelvio 25, 23100 Sondrio, Italy*

*(Received 4 November 1998; Revised 10 March 1999; In final form 5 April 1999)*

**The best treatment of bile duct lithiasis in the era of the laparoscopic cholecystectomy has not yet been defined, as we can choose between different kinds of therapies which range from the traditional laparotomic treatment to the more modern endoscopic and laparoscopic techniques. Although in some centres a wholly laparoscopic access to the bile duct is used, we believe that the fastest, safest and most reliable method is still the sequential treatment, endoscopic and laparoscopic. In our experience the endoscopic cleaning of the choledochus was performed in 87.1% of the cases, with no mortality and 4.2% morbidity.**

*Keywords:* Bile duct stones, Endoscopic sphincterotomy, ERCP, Laparoscopic cholecystectomy

### INTRODUCTION

On average we would expect 5–15% of patients suffering from gallstones to have calculosis present in the choledochus, with an increase in incidence parallel to increase in age [1,2,3]. These are the vast majority of secondary cases of migration through the cystic duct of calculosis present in the gallbladder, and may give rise to various clinical conditions:

- complicated (obstructive jaundice, cholangitis, dysfunction of the sphincter of Oddi, acute pancreatitis),
- sub-clinical obstruction (elevation of hepatic enzymes or dilation of the choledochus shown by echographia),
- unexpected (discovered by cholangiography),
- post-cholecystectomy.

Endoscopic sphincterotomy (ES) has undoubtedly revolutionised the therapeutic approach to bile duct lithiasis; initially used only in the case of residual or recurrent calculosis after cholecistectomy, its subscriptions then grew and it has become the most widely used method. In the same way, mini-invasive laparoscopic surgery has not only found its fullest application in the case of cholecystectomy,

* Corresponding author. Tel.: 0342521111. Fax: 0342214004.

but it has also permitted new and innovative surgical solutions for the treatment of calculosis of the choledochus. If we exclude cases of lithiasis in patients with previous cholecystectomy, in which ES is the preferred method, today for the treatment of gallstones and bile duct stones various therapeutic options exist:

- Removal of the stones using the traditional laparotomic way.
- Sequential treatment: pre-operative ES followed by laparocholecystectomy (LC).
- "Inverse" sequential treatment: post-operative LC + ES.
- Simultaneous treatment: intra-operative LC + ES.
- Laparoscopic excision "in one time".

In this paper we will report our experience regarding the treatment of bile duct lithiasis associated with gallstones.

### MATERIALS AND METHODS

During the period from December 1991 to August 1998, at the General Surgery Ward of Sondrio Hospital, 751 patients suffering from symptomatic gallstones underwent laparoscopic cholecystectomy. Whereas initially the patients with an associated pathology regarding the choledochus were treated with traditional surgical methods via laparotomy, after October 1992 we began a combined endoscopic and laparoscopic treatment. In the pre-operative phase we identified all those patients with suspected associated bile duct stones, who therefore underwent ERCP (Endoscopic Retrograde Cholangio-Pancreatography). The choice of these patients was based on a correct case history and medical history (jaundice, cholangitis or pancreatitis), the elevation of hepatic enzymes, the dilation of the choledochus, with or without presence of stones, shown by echographia or cholangiography IV, although this last method for the moment is no longer available.

ERCP has always been performed regardless of the age or general conditions of the patient, as it is considered less dangerous than a surgical operation under general anaesthetic: in the case of a serious respiratory insufficiency we administer low-flow oxygen during the examination, while in the case of serious coagulopathy, coagulation factors are administered in infusion before the examination; furthermore we use an ultra-short term antibiotic prophylaxis with cephalsporins and pharmacological sedation based on IM meperidine and IV diazepam. If a calculosis or a dilation of the choledochus is discovered during the retrograde colangiography, we proceed immediately with an ES, which is often performed anyway only on the basis of a suspicion, frequently confirmed by the section of the papilla, by a microlithiasis which not even the cholangiography is able to pick up.

Even if many stones could pass spontaneously after a standard ES, especially those with a diameter of less than 1 cm, we always explore the biliary tract with the Dormia basket or the Fogarty ball, checking afterwards by means of a cholangiography that the removal has taken place. If the stones present are too large, too many or impacted, or if there is a stenosis of the terminal choldochus or an unfavourable duodenopapillary anatomy which make instrumental extraction difficult, we occasionally resort to complementary endoscopic methods such as the positioning of naso-biliary tubes or of prostheses, whereas more often we opt for traditional surgery. We are not equipped with more sophisticated methods such as extra- or intracorporeal lithodialysis, which could increase the success rate of the ES. The aim of our work is to verify the results obtained by associating the endoscopic methods with the laparoscopic ones. The average age of patients subjected to ES was 65.5 years (range 24–94). Their follow-up period ranges from 1 to 70 months.

### RESULTS

In 312 patients the pre-operative investigations suggested the presence of bile duct stones and therefore the use of ERCP was indicated. Cannulating the papilla was impossible in 4 patients (1.2%). ERCP alone involved 5 cases of complications (1.6%): 3 duodenal micro-perforations, due to the presence of a large bulbar ulcer in one case, and in two cases due to a paravaterian diverticulum, manifested with retropneumoperitoneum and pneumomediastinum, which were rapidly solved with medical therapy (antibiotics, NPT, naso-gastric tubes) and later the patients underwent laparocholecystectomy; in two other cases a large oedema on the duodenal wall caused a laceration of the duodenum with peritonitis and subsequent surgical treatment of stitching and drainage. 41.9% of the patients (131) had a normal cholangiographic history, while 177 of them were subjected to ES for biliary calculosis or sand (135) or dilation of the choledochus without evident stones, but in keeping with benign stenosis of the sphincter of Oddi or biliary pancreatitis (42).

ES was performed in 168 cases, while in 9 cases (5%) it was impossible to carry out, either because of the presence of a calculus impacted in the papilla (6 cases) or because of an anatomic alteration of the duodenum due to the presence of a bilio-digestive fistula (2 cases), or because of a compact stenosis of the terminal choldochus which even prevented its being probed intra-operatively by means of choledochotomy. The extraction of the stones was not completely successful in 14 cases (7.9%): 4 because of empiercement of the bile duct, 2 because of a discrepancy between the diameter of the calculus and that of the sphincterotomy, one due to a stenosis of the terminal part of the bile duct, while in seven cases it was impossible to seize them with the basket or the ball. In another 4 cases, the stones were left where they were because it was impossible to extract them came out spontaneously, or after choledochus lavage, by means of the naso-biliary tube, whereas in another case involving stenosis of the distal tract of the bile duct, a biliary prosthesis was placed in position thus normalising the hemato-chemical examinations. Therefore, in synthesis, the endoscopic clearance of the choledochus was not successful in 23 cases (12.9%).

The morbidity of ES was 4.2% (7 cases): 6 cases of bleeding (3.6%), of whom two required blood transfusions (one patient was cirrhotic with PT of 25% and serious general conditions which contraindicated surgical intervention), one required a surgical operarion to apply 2 hematostatic points, while the others were treated with adrenaline injections, tamponage with the ball or current application with the sphincterotomus. One case of clinically evident pancreatitis (0.6%) was easily treated with simple medical therapy. There were no cases of mortality either for the ERCP or for the endoscopic sphincterotomy. The diagnostic and therapeutic data regarding the endoscopy is summarised in Table I.

Of the 154 remaining patients, 104 underwent combined endoscopic and laparoscopic treatment: 101 sequential, with pre-operative ES and LC, one simultaneous treatment (with intra-operative LC+ES) and two underwent "inverse" sequential treatment which was made necessary due to the occurrence of cholestatic jaundice in the period immediately following the laparoscopic cholecystectomy, which required post-operative ES. In 20

TABLE I Results of diagnostic and therapeutic endoscopy

| ERCP | | Endoscopic sphincterotomy | |
|---|---|---|---|
| Total number 312 | | Total number 177 | |
| Failure 11.4 (1.2%) | | Failure | n. 23 (12.9%) |
| | | failed sphincterotomy | n. 9 (5.0%) |
| | | inability clearance common bile duct | n. 14 (7.9%) |
| Normal radiology | n. 131 (42.0%) | Successes | n. 154 (87.1%) |
| Pathologic radiology | n. 177 (56.8%) | | |
| stones/sludge in the CBD | n. 135 (43.2%) | | |
| stenosis papilla/pacreatitis | n. 42 (13.6%) | | |
| Complications | n. 5 (1.6%) | Complications n. 7 (4.2%) | |

TABLE II Patients undergoing endoscopic and laparascopic treatment, with successful CBD clearance

| | Sequential treatment (ES pre-operative + LC) | Simultaneous treatment (LC + ES intra-operative) | Opposite sequential treatment (LC + ES post-operative) | ES + cholecystis "*in situ*" | ES in patients with previous cholecystectomy |
|---|---|---|---|---|---|
| Stones/sludge in the CBD | 75 | 1 | 0 | 14 | 23 |
| Stenosis of the papilla | 11 | 0 | 1 | 5 | 6 |
| Gallstones pancreatitis | 15 | 0 | 1 | 1 | 1 |
| Total | 101 | 1 | 2 | 20 | 30 |

cases the cholecyst was left *in situ*, considering that the patients were very advanced in age or in poor general conditions. Finally, in 30 patients who had already undergone cholecystectomy, the endoscopic papillotomy allowed us to decisively treat the pathology of the choledochus. The summarising data is shown in Table II.

The interval between the ES and the LC was on average 6 days (range 1–13). The pre-operative endoscopic approach did not make the laparoscopic treatment more difficult, seeing that there was no sign of secondary inflammation nor did it modify the duration of the post-operative period of hospitalisation or the mortality or morbidity rate compared to single laparoscopic cholecystectomies. In particular, the conversion rate to laparotomy was 3.8%, also considering the fact that there are no selection criteria for laparoscopic operations. We did not observe any long term complications after ES and cholecystectomy, whereas in the patients treated only with ES, leaving the cholecyst *in situ*, complications arose in 3 cases, equal to 15%. Two of these, who were discharged with hemato-chemical examinations and control cholangiography within normal ranges, again presented calculosis of the bile duct after 20 days and 13 months respectively, while another patient underwent a laparocholecystectomy for recurrent biliary colic after 29 months.

### DISCUSSION

Endoscopy and mini-invasive surgery have radically updated the treatment of bile duct lithiasis in all its various manifestations, which up until only a few years ago was treated exclusively in the traditional surgical way. At the moment several therapeutic options are available, which include, as well as the traditional laparotomy, endoscopic and laparoscopic techniques which can be combined in different ways insofar as the sequence of the various manoeuvres is concerned. The choice is made on the basis of the operator's experience, as long as he has the necessary training, equipment and instruments for the endoscopic and laparoscopic treatment.

The method most frequently used is the sequential one – pre-operative ES followed by LC [3–5]. ES is a method which has been in use for over 20 years, and by now its validity and safety are well known, with a mortality rate of 0.3–2% [1,3,6–8] and a morbidity of 4–8% [1,3,6,7]. The clearance of the choldochus is achieved in 85–90% of cases, which can reach 97% when associated with methods of lithodialysis [3,9]. The advantage of laparoscopic cholecystectomy is the greater acceptance from the patient, and with the sequential therapy we can add a shorter post-operative period in hospital, the aesthetic aspect and the advantages of the endoscopic approach, which are: it is simple to carry out, it is therapeutically effective, and the rate of complications and mortality is low. If performed quickly and using the correct pharmacological sedation, the endoscopic treatment can be tolerated well even by elderly patients in poor physical conditions. However, of fundamental importance for the combined endoscopic and surgical treatment is a thorough endoscopic experience, which can guarantee a high success rate and a low incidence of complications, as well as good surgical facilities, to be able to face any complications which might arise. The patients

(10–15%) who underwent ES, but with the cholecyst left *in situ*, present complications at the level of the cholecyst, because of which it is desirable to always perform the cholecystectomy [3] if there are no serious contraindications.

The most recent and modern approach is the excision of the bile duct stones in one laparoscopic time, by trans-cystic access or by performing a more complicated, but certainly more effective, choledochotomy. This is advantageous because it avoids the risks of endoscopic papillotomy, leaving the papilla integral, and of course the laparotomy is not performed. However, it has a higher rate of complications, and limits such as massive calculosis of the bile duct, choledochus not dilated, the presence of stones in that area and an unfavourable anatomy of the gallbladder (long, narrow, medial insertion, difficult to probe) [6,10]. In expert hands a success rate of 80–95% can be reached [1,3,10,11], but for the moment, however, this approach is not yet well established, since it can be applied only in a limited number of patients and requires operator experience, sophisticated instrumentation and additional skills, such as pre-operative suturing or endoscopy. The wholly laparoscopic treatment may be the necessary choice, if the endoscopic papillotomy is unsuccessful or if bile duct stones are diagnosed during surgery.

The treatment in one time, ES and LC, is a method half-way between the two previous ones, which has the advantage of excellent patient compliance, and could also theoretically involve a higher possibility of ERCP-ES success by using a combined manoeuvre, by inserting a guiding thread through the trans-cystic or trans-papillary way. However it is more difficult for the endoscope operator to carry out the examination with the patient in the dorsal position, it involves longer general anaesthesia and the air insufflated with the endoscope could possibly hinder the laparoscopic manoeuvres. The "inverse" sequential treatment (first laparocholecystectomy, then post-operative ES) is a possible choice, making it unnecessary to attempt wholly laparoscopic treatment, or in the case that this treatment is not successful. However, if post-operative endoscopic treatment failed, the patient would undergo three types of operation: laparoscopic, endoscopic and finally laparotomic, and so for this reason it is not universally accepted.

In order to programme a correct therapy of bile duct lithiasis, the crucial point is the identification of patients "at risk" of calculosis of the choledochus. In the therapeutic strategy which we have adopted, showed in Fig. 1, the diagnosis is always made in the pre-operative phase with a methodical selection of patients based on the case history and medical history, laboratory exams and the echography. The ERCP is only used in cases chosen for suspected bile duct stones, and not as a routine diagnostic examination. In fact, although it is the most diagnostically sensitive method, it is still an invasive method with a certain morbidity rate (2–3%) and a mortality rate of 0.2% [3,8,12,13]. If the pre-operative study is negative, we carry out the simple laparoscopic cholecystectomy without performing an intra-operative cholangiography, seeing that the probability of bile duct lithiasis not being picked up in the pre-operative diagnostic phase does not exceed 3% [3,14]. Furthermore, in our opinion, this does not eliminate the possibility of iatrogenic lesions of the biliary tract which may be caused by cannulation of the cystic [3]. For to avoid intra-operative complication, careful laparoscopic dissection and clear demonstration of the anatomy are mandatory, when there is uncertainty about the anatomy we prefer to convert to open cholecystectomy.

We generally carry out a very wide sphincterotomy, within the limits of the local anatomy, of the degree of dilation of the bile duct and of the dimensions of the stones to be excised, which can therefore allow good biliary defluvium, thus reducing the incidence of cholangitis or pancreatitis, and which facilitates the expulsion of the stones, both spontaneously and with instrumental methods. The wideness of the sphincterotomy probably also prevents long term complications, seeing that we have not observed any cases of stenosis of the papilla or recurring lithiasics.

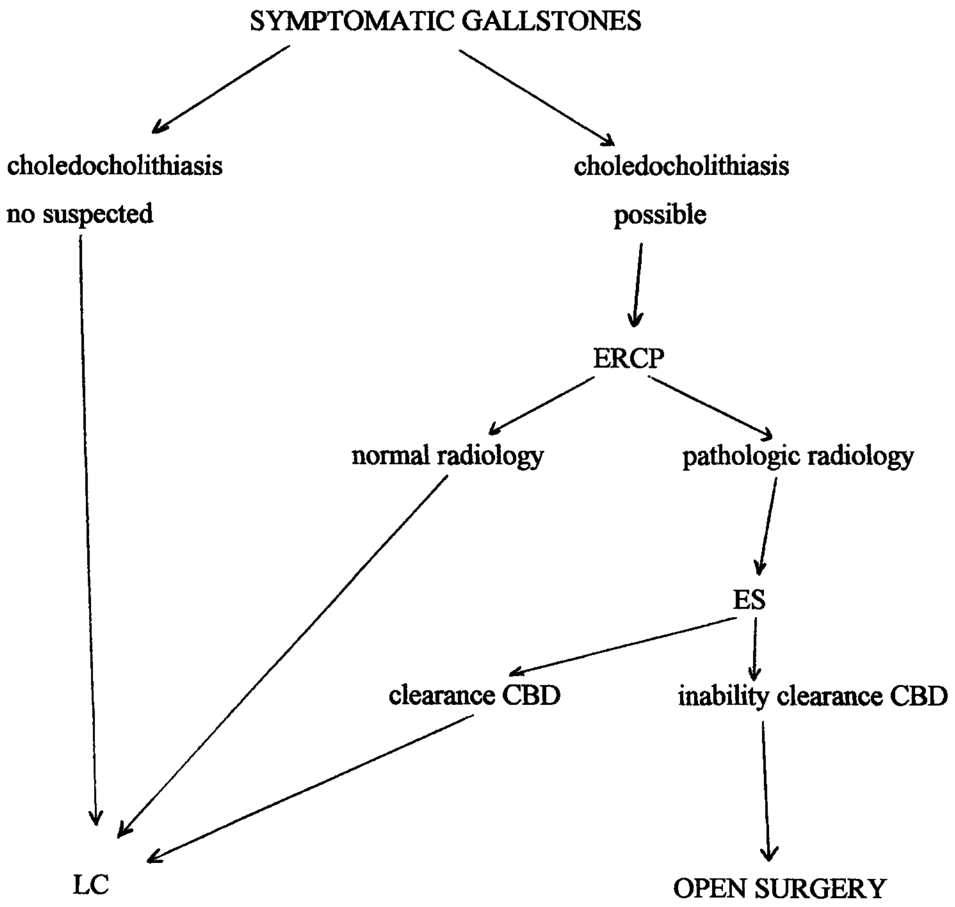

FIGURE 1 Algorithm for the management of patients with symptomatic gallstones (LC, laparocholecystectomy; ERCP, endoscopic retrograde cholangio-pancreatography; ES, endoscopic sphincterotomy; CBD, common bile duct).

We do not believe that young age must be a contraindication to ES, seeing the lack of delayed sequences on the Oddi; even the tenuous biliary tract creates no problems for us, because although it is true that the rate of complications may rise, these complications are certainly just as frequent in open surgery or in "wholly laparoscopic" surgery.

### *References*

[1] Paganini, A., Feliciotti, F., Chan, R., Guerrieri, M., Di Emiddio, M., Campagnacci, R., Berti, F. and Lezoche, E. Laparoscopic access to the common bile duct, Atti del convegno dell'European Association of Surgical Sciences, Ischia 1994: pp. 126–129.

[2] McEntee, G., Grace, P.A. and Bouchier-Hayes, D. Laparoscopic cholecystectomy and the common bile duct. *Br. J. Surg.* 1991; **78**: 385–386.

[3] Perissat, J., Huibregste, K., Keane, F.B.V., Russel, R.C.G. and Neoptolemos, J.P. Management of bile duct stones in the era of laparoscopic cholecystectomy. *Br. J. Surg.* 1994; **81**: 799–810.

[4] Brodisch, R.J. and Fink, A.S. ERCP, cholangiography and laparoscopic cholecystectomy. The Society of American Gastrointestinal Endoscopic Surgeons (SAGES) opinion survey. *Surg. Endosc.* 1993; **7**: 3–8.

[5] Arnaud, J.P., Tuech, J.J., Cattan, F., Casa, C. and Bergamaschi, R. Endoscopici sphincterotomy prior to laparoscopic cholecystectomy for the treatment of cholelitiasis. *Br. J. Surg.* 1997; **84**(Suppl. 2): 5–6.

[6] Binmoeller, K.F. Endoscopic management of biliary duct stones. Atti del convegno dell'European Association of Surgical Sciences, Ischia 1994; pp. 112–117.

[7] Conigliaro, R., Ricci, E., Orsi, P., Pacchione, D., Mortilla, M.G., Bertoni, G., Sassatelli, R., Olivetti, G. and Bedogni, G. Il trattamento sequenziale della calcolosi colecisto-coledocica. *Giorn. Ital. End. Dig.* 1993; **16**: 63–72.

[8] Rijna, H., Borgstein, P.J., Meuwissen, S.G.M., de Btauw, L.M., Wildenborg, N.P. and Cuesta, M.A. Selective preoperative endoscopic retrograde cholangiopancreatography in laparoscopic biliary surgery. *Br. J. Surg.* 1995; **82**: 1130–1133.

[9] Widdison, A.L., Longstaff, A.J. and Armstrong, C.P. Combined laparoscopic and endoscopic treatment of gallstones and bile duct stones: a prospective study. *Br. J. Surg.* 1994; **81**: 595–597.
[10] Cuschieri, A. and Berci, G. Laparoscopic treatment of common duct stones. In: *Laparoscopic Biliary Surgery*. Chap. 9. Oxford: Blackwell Scientific Publications, 1992.
[11] Rhodes, M., Nathanson, L., O'Rourke, N. and Fielding, G. Laparoscopic exploration of the common bile duct: lessons learned from 129 consecutive cases. *Br. J. Surg.* 1995; **82**: 666–668.
[12] Graham, S.M., Flowers, J.L., Scott, T.R., Bailey, R.W., Scovill, W.A., Zucker, K.A. and Imbembo, A.L. Laparoscopic cholecystectomy and common bile duct stones. The utility of planned perioperative endoscopic retrograde cholangiography and sphincterotomy: experience with 63 patients. *Ann. Surg.* 1993; **218**(1): 61–67.
[13] Voyles, C.R., Sanders, D.L. and Hogan, R. Common bile duct evaluation in the era of laparoscopic cholecystectomy. 1050 casese later. *Ann. Surg.* 1994; **219**(6): 744–752.
[14] Mitchell, S.A., Jacyna, M.R. and Chadwick, S. Common bile duct stones: a controversy revisited. *Br. J. Surg.* 1993; **80**: 759–760.

